# In Vitro Antimicrobial Potential of Portuguese Propolis Extracts from Gerês against Pathogenic Microorganisms

**DOI:** 10.3390/antibiotics13070655

**Published:** 2024-07-16

**Authors:** Rafaela Dias Oliveira, Carina Araújo, Cristina Almeida-Aguiar

**Affiliations:** 1 Life and Health Sciences Research Institute (ICVS), University of Minho, 4710-057 Braga, Portugal; rafaeladiasoliveira98@gmail.com; 2 ICVS/3B’s—PT Government Associate Laboratory, 4710-057 Braga, Portugal; 3 Biology Department, University of Minho, 4710-057 Braga, Portugal; sacarinaaraujo@gmail.com; 4 CBMA—Centre of Molecular and Environmental Biology, University of Minho, 4710-057 Braga, Portugal

**Keywords:** Portuguese propolis, antimicrobial activity, pathogenic microorganisms, antibiotic resistance

## Abstract

Antimicrobial resistance (AMR) is one of humanity’s main health problems today. Despite all the breakthroughs and research over the past few years, the number of microbial illnesses that are resistant to the available antibiotics is increasing at an alarming rate. In this article, we estimated the biomedical potential of Portuguese propolis harvested from the Gerês apiary over five years, evaluating the in vitro antimicrobial effect of five hydroalcoholic extracts prepared from five single propolis samples and of a hydroalcoholic extract obtained from the mixture of all samples. The antimicrobial potential was firstly assessed by determining the minimum inhibitory concentration (MIC) of these extracts against a panel of three Gram-positive (*Bacillus subtilis*, methicillin-sensitive *Staphylococcus aureus,* and methicillin-resistant *Staphylococcus aureus*) and one Gram-negative bacteria (*Escherichia coli),* as well as two yeasts (*Candida albicans* and *Saccharomyces cerevisiae*). As MIC values against each bacterium were consistent across all the evaluated propolis extracts, we decided to further conduct a disk diffusion assay, which included three commercial antibiotics—erythromycin, vancomycin, and amoxicillin/clavulanic acid—for comparison purposes. In addition to displaying a concentration-dependent antibacterial effect, the hydroalcoholic extracts prepared with 70% ethanol exhibited stronger antimicrobial capacity than vancomycin against *B. subtilis* (% of increase ranged between 26 and 59%) and methicillin-sensitive *S. aureus* (% of increase ranged between 63 and 77%). Moreover, methicillin-resistant *S. aureus* (MRSA) showed susceptibility to the activity of the same extracts and resistance to all tested antibiotics. These findings support that propolis from Gerês is a promising natural product with promising antimicrobial activity, representing a very stimulating result considering the actual problem with AMR.

## 1. Introduction

Antimicrobial resistance (AMR)—defined as the capacity of a microorganism to survive exposure to a defined dose of an antimicrobial agent—is one of the major current threats to public health [1,2]. Despite all the advancements and research over the past years, the number of microbial illnesses that are resistant to all known and approved antibiotics is increasing at an alarming rate [3]. This problem is exacerbated by the extension of resistance from the restricted hospital environments into open communities and by the ongoing development of novel resistance mechanisms [4]. In 2019, 4.95 million deaths caused by AMR were recorded, among which 1.27 million were caused by bacterial resistance [5]. According to the Organization for Economic Cooperation and Development, infections with resistant microorganisms will cause 2.4 million deaths in Europe, North America, and Australia over the following 30 years [6]. In fact, it is predicted that Southern Europe will have the highest mortality rate and will be the most affected region worldwide [2,6]. In short, the current pattern of increased resistance of microbial pathogens to the available drugs has stimulated the introduction of new alternative agents in the treatment of infectious diseases. Thus, the interest in researching natural products, such as propolis and its isolated compounds, as antimicrobial agents has emerged.

Propolis, a natural resinous product produced by honeybees (mainly *Apis mellifera* L.) is a mixture of resins collected from plant exudates which are digested with ß-glucosidase and mixed with pollen and waxes to form the final product [7,8,9]. The chemical composition of this natural product is extremely complex and highly variable, depending on distinct factors, such as the bee species, surrounding flora, local climate, and geographic location. In fact, about 800 compounds were already identified in propolis [10]. These compounds belong to distinct classes, namely, phenolic acids (cinnamic and caffeic acids) and their esters, flavonoids (flavones, flavanones, flavonols, and dihydroflavonols chalcones), and terpenes [9,11,12,13]. Over the years, several biological properties of this natural product have been confirmed, including its antimicrobial capacity, which has been linked to the presence of polyphenolic compounds—namely, flavonoids (chrysin, galangin, pinobanksin, pinocembrin), phenolic acids (caffeic, cinnamic, and ferulic acids) and their esters [7,9,14], and terpenes [9,15]. Notably, some of these propolis compounds, such as chrysin or apigenin, for example, have been shown not only to exhibit antimicrobial effects against microorganisms but also to potentiate the activity of antibiotics [16,17]. According to published data, propolis is a really powerful antibacterial agent that is more effective against Gram-positive bacteria than against Gram-negative bacteria [9,18], and has bactericidal effects which are correlated with several mechanisms, including the inhibition of cell division and protein synthesis, the destruction of cell walls and bacterial cytoplasm, and the inhibition of bacterial mobility and enzyme activity [19,20]. Additionally, propolis displays bacteriostatic activity against diverse bacterial genera [18,21].

Portuguese propolis from Gerês is one of the most studied in Portugal [13,22,23,24,25,26,27,28,29]. Recent results revealed potent antimicrobial and antioxidant activities, regardless of the harvesting year—which seems to be a distinctive and unique feature of this propolis. Indeed, this constancy was also observed in the chemical profile of this national product [22]. Moreover, Peixoto et al. [28] reported that mixtures of propolis samples from Gerês harvested in different years are equally or more effective than the single extracts. This highlights a possible step toward standardization since the combinations of fewer and more active extracts result in similar bioactivities. The use of propolis mixtures is also important to ensure propolis availability, as well as to value this national natural resource, allowing propolis application in food, cosmetic, and medical areas.

Research on Portuguese propolis increased the potential for generating novel and bioactive propolis-based formulations [25,30]. A prior study conducted by Araújo et al. [29] demonstrated the anti-inflammatory capacity of Portuguese propolis from Gerês, both in vivo and in vitro, using single extracts and extracts of mixtures of propolis samples. In the present study, our main goal was to evaluate the antimicrobial effect of the same extracts of Portuguese propolis from Gerês previously studied by Araújo et al. [29]. More precisely, we aimed to assess their effectiveness as antimicrobial agents against a selected panel of susceptibility indicator strains and to compare such effectiveness with the action of three commercial antibiotics—amoxicillin/clavulanic acid (AMC), erythromycin (ERY), and vancomycin (VAN).

## 2. Results

### 2.1. In Vitro Antimicrobial Potential of Propolis from Gerês

Globally, none of the six extracts was active against yeast, *Escherichia coli*, and methicillin-resistant *Staphylococcus aureus* at the highest tested concentration in the agar dilution assays (Table 1). However, the same hydroalcoholic extracts were found to be active against *Bacillus subtilis* and methicillin-sensitive *Staphylococcus aureus*. No differences were observed in the antimicrobial capacity of the different extracts against each of the tested bacteria, as indicated by identical MIC values (Table 1).

Since no differences were observed across the antimicrobial activity of the different hydroalcoholic extracts, we decided to pursue a disk diffusion assay to try to distinguish the capacity of each extract against the susceptible panel of microorganisms. Therefore, we evaluated the antimicrobial potential of the hydroalcoholic extracts of propolis from Gerês, either from single samples (G.EE_70_) or from the resultant mixtures (mG.EE_70_) (Supplementary Table S1). Additionally, we tested the extracts obtained by diluting the G.EE70 samples (G11.EE35, G12.EE35, G13.EE35, G14.EE35, G15.EE35, and mG.EE35) to assess the effect of dilution on the antimicrobial potential of the propolis extracts. Globally, none of the analyzed extracts was active against yeasts as no growth inhibition zones were observed. On the other hand, antibacterial activity was detected against all the tested bacteria strains, and the G.EE_70_ group displayed invariably inhibition zones with wider diameters than the ones observed within the G.EE_35_ group.

#### 2.1.1. Antimicrobial Effect of Propolis Extracts against *Bacillus subtilis*

**Figure 1:**
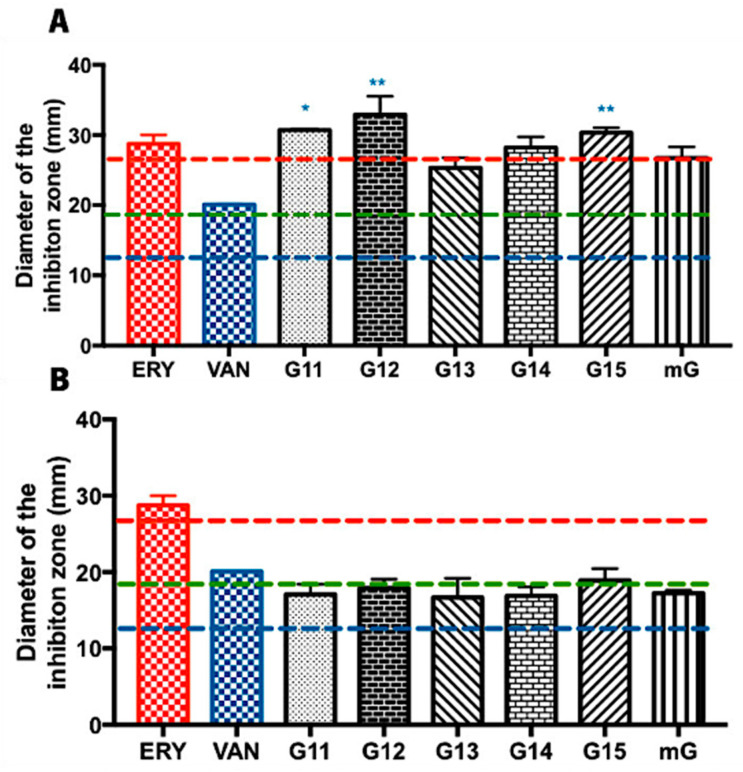
Antibacterial effect of the Gerês propolis hydroalcoholic extracts against *Bacillus subtilis*. Diameter of the inhibition zones displayed by (**A**) G.EE_70_ samples, erythromycin, and vancomycin and by (**B**) G.EE_35_ samples, erythromycin, and vancomycin. Results are expressed as means ± SD (* 0.05 > *p* ≥ 0.01; ** 0.01 > *p* ≥ 0.001). No growth inhibition zones were observed for disks containing ethanol 70% or ethanol 35%. Reference values of inhibition zone diameters above which a microorganism is susceptible to ERY, AMC, and VAN [33] are represented with red, green, and blue dashed lines, respectively. (ERY: erythromycin; AMC: amoxicillin/Clavulanic Acid; VAN: vancomycin; G: Gerês, mG: mixture of propolis samples from Gerês).

The antimicrobial potential of propolis hydroalcoholic extracts from Gerês was evaluated against *Bacillus subtilis*, often used as a Gram-positive model organism [31,32]. Overall, the inhibition zone diameters displayed by the G.EE_70_ samples were generally higher (values ranging from 25.25 ± 2.46 to 31.92 ± 4.48 mm) (Figure 1A) than the ones displayed by the G.EE_35_ samples (values ranging between 16.67 ± 3.62 and 19.92 ± 2.72 mm) (Figure 1B). Additionally, and considering the Zone Diameter Interpretive Standards for each antibiotic–microorganism [33], this species seems to be more susceptible to the antibiotics ERY and VAN and resistant to AMC (Supplementary Table S1 and Figure 1).

The antimicrobial potential did not vary significantly between samples harvested in different years (ANOVA_1w_, F_(5,12)_ = 2.793, *p* = 0.0673; Figure 1A; ANOVA_1w_, F_(5,12)_ = 0.755, *p* = 0.05984; Figure 1B), suggesting a constancy in antibacterial activity against *Bacillus subtilis*. When comparing with the tested antibiotics, however, the post hoc analysis for the G.EE_70_ group (ANOVA_1w_, F_(7,14)_ = 5.98, *p* = 0.002; Figure 1A) showed significant differences between (i) G11.EE and VAN; (ii) G12.EE and VAN; and (iii) G15.EE and VAN. Regarding the G.EE_35_ group, no significant differences were observed across the inhibition zone diameters displayed by propolis extracts and the tested antibiotics (ANOVA_1w_, F_(6,12)_ = 0.78, *p* = 0.60; Figure 1B). In fact, the findings point to the limited antibacterial action of G.EE_35_ samples against the tested microorganism. Thus, in summary, G11.EE_70_, G12.EE_70_, and G15.EE_70_ have the highest antibacterial activity against *Bacillus subtilis* and are a feasible and potential alternative to the tested antibiotics.

#### 2.1.2. Antimicrobial Effect of Propolis Extracts against Methicillin-Sensitive *Staphylococcus aureus*

**Figure 2:**
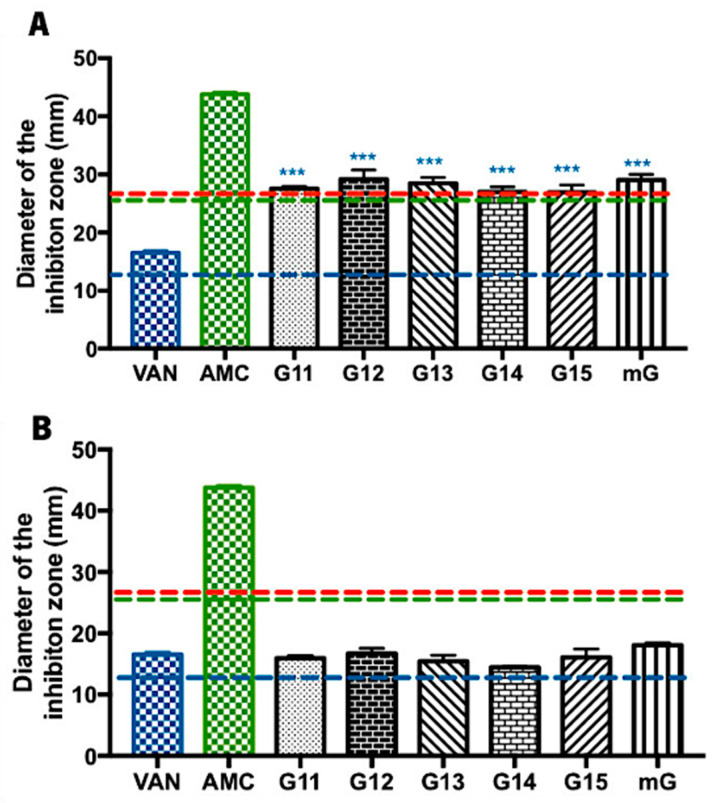
Antibacterial effect of Gerês propolis hydroalcoholic extracts against methicillin-sensitive *Staphylococcus aureus*. Diameter of the inhibition zones displayed by (**A**) G.EE_70_ samples, Vancomycin, and Amoxicillin/Clavulanic acid and by (**B**) G.EE_35_ samples, Vancomycin, and Amoxicillin/Clavulanic acid. Results are expressed as means ± SD (*** *p* < 0.001). No growth inhibition zones were observed for disks containing ethanol 70% or ethanol 35%. Reference values of inhibition zone diameters above which *Staphylococcus* spp. is susceptible to ERY, AMC, and VAN [33] are represented with red, green, and blue dashed lines, respectively. (ERY: erythromycin; AMC: amoxicillin/Clavulanic Acid; VAN: vancomycin; G: Gerês, mG: mixture of propolis samples from Gerês).

Methicillin-sensitive *Staphylococcus aureus* is a Gram-positive bacterium that is pathogenic for humans, causing soft (e.g., furuncles and cellulitis) and very serious infections (e.g., blood stream infections and pneumonia) [34]. Regarding the evaluation of the Gerês propolis hydroalcoholic extracts’ antibacterial capacity against *Staphylococcus aureus*, G.EE_70_ samples exhibited considerably higher inhibition zones (values ranging from 26.93 ± 2.20 to 29.17 ± 2.75 mm) (Figure 2A) than the ones displayed by the G.Ee_35_ samples (values ranging from 14.42 ± 0.14 to 18.08 ± 0.62 mm) (Figure 2B). Considering the reference values of the inhibition zone diameters for the tested antibiotics and *Staphylococcus* spp. [33], this MSSA strain was revealed to be susceptible to the action of both VAN and AMC and resistant to ERY (Supplementary Table S1 and Figure 2).

The antimicrobial potential of hydroalcoholic extracts of propolis from Gerês against *S. aureus* is consistent and independent of the harvesting year (ANOVA_1w_, F_(5,12)_ = 0.877, *p* = 0.5248; Figure 1A; ANOVA_1w_, F_(5,12)_ = 1.547, *p* = 0.2478; Figure 1B). Nevertheless, the G.EE_70_ group post hoc analysis (ANOVA_1w_, F_(7,16)_ = 5.98, *p <* 0.001; Figure 2A) revealed significant differences across (i) G11.EE_70_ and VAN; (ii) G12.EE_70_ and VAN; (iii) G13.EE_70_ and VAN; (iv) G14.EE_70_ and VAN; (v) G15.EE_70_ and VAN; and (vi) mG.EE_70_ and VAN. These results suggest the G.EE_70_ samples have stronger antibacterial capacity than vancomycin. In summary, in the G.EE_70_ group, all extracts showed antibacterial activity effectiveness, and the mG.EE_70_ exhibited higher capacity than almost all the individual extracts, indicating that blending propolis with either a high or low antibacterial capacity may enhance this bioactivity.

Regarding G.EE_35_ samples, significant differences were not observed among the extracts (Figure 2B), and the diameters of the inhibition zones displayed were lower than the ones obtained with the AMC antibiotic. However, they were remarkably similar to the inhibition diameter produced by VAN, and, even though no statistically significant differences were observed (ANOVA_1w_, F_(6,13)_ = 2.37, *p* = 0.09; Figure 2B), mG.EE_35_ could be a good option when a low ethanol concentration is required because it is slightly more active than individual extracts (Supplementary Table S1 and Figure 2B).

#### 2.1.3. Antimicrobial Effect of Propolis Extracts against Methicillin-Resistant *Staphylococcus aureus*

**Figure 3:**
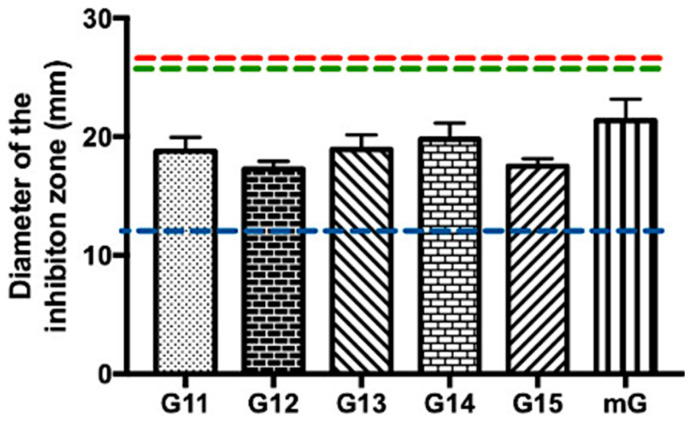
Antibacterial effect of Gerês propolis hydroalcoholic extracts against methicillin-resistant *Staphylococcus aureus*. Diameter of the inhibition zones displayed by G.EE_70_ samples. Results are expressed as means ± SD. No growth inhibition zones were observed for disks containing ethanol 70%. Reference values of inhibition zone diameters above which *Staphylococcus* spp. is susceptible to ERY, AMC, and VAN [33] are represented with red, green, and blue dashed lines, respectively. (ERY: erythromycin; AMC: amoxicillin/Clavulanic Acid; VAN: vancomycin; G: Gerês, mG: mixture of propolis samples from Gerês).

Methicillin-resistant *S. aureus* is currently recognized as one of the major multidrug-resistant bacterial pathogens, posing a significant threat to human health [35,36,37]. Considering the need for new antibacterial compounds to overcome this methicillin resistance, Gerês propolis hydroalcoholic extracts were tested against MRSA (Supplementary Table S1 and Figure 3).

In general, the tested commercial antibiotics (ERY, VAN, and AMC) had no inhibitory effect against MRSA, demonstrating the already known resistance of this bacterium to these antibiotics (Supplementary Table S1). No antibacterial effect was observed after treatment with the hydroalcoholic extracts of Gerês propolis containing ethanol 35% either (Supplementary Table S1). Regarding the G.EE_70_ group, all the tested G.EEs/mG.EEs caused an inhibitory effect in the growth of MRSA, with values ranging between 17.25 ± 1.15 and 21.33 ± 3.17 mm (Supplementary Table S1 and Figure 3). In fact, the antimicrobial potential of these extracts did not vary significantly between samples harvested in different years (ANOVA_1w_, F_(5,12)_ = 1.51, *p* = 0.26; Figure 3), revealing a constancy in the antibacterial potential against MRSA. Curiously, the G.EE_70_ group displayed inhibition halos even larger than the reference inhibition zone diameter for vancomycin against *Staphylococcus* spp. (blue line, Figure 3). Although not significantly different, the extract of propolis mixtures—mG.EE_70_—seemed to be the most effective extract against this bacterium.

#### 2.1.4. Antimicrobial Effect of Propolis Extracts against *Escherichia coli*

**Figure 4:**
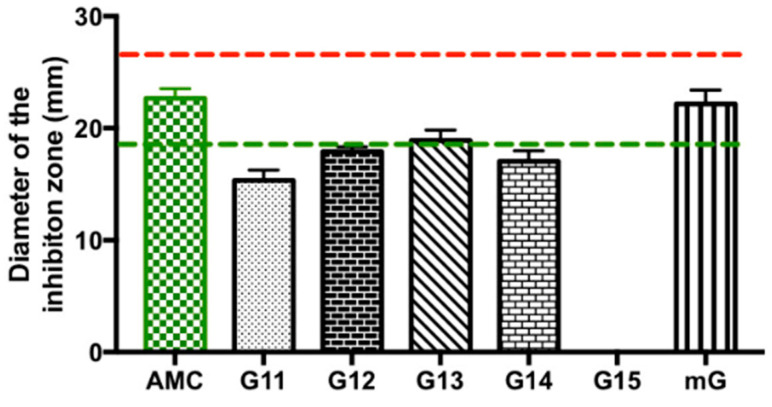
Antibacterial effect of Gerês propolis hydroalcoholic extracts against *Escherichia coli*. Diameter of the inhibition zones displayed by G.EE_70_ samples and AMC. Results are expressed as means ± SD. No growth inhibition zones were observed for disks containing ethanol 70%. Reference values of inhibition zone diameters above which a microorganism is susceptible to ERY and AMC are represented with red and green dashed lines, respectively [33]. Vancomycin has no reference diameter against Gram-negative bacteria because of its inability to breach the outer membrane barrier [40]. (ERY: erythromycin; AMC: amoxicillin/Clavulanic Acid; G: Gerês, mG: mixture of propolis samples from Gerês).

Lastly, the antimicrobial efficacy of Gerês propolis hydroalcoholic extracts was investigated against *Escherichia coli* (*E. coli*), a Gram-negative bacillus commonly found in the intestinal tract of humans and animals [38]. Overall, *E. coli* was only susceptible to the action of the G.EE_70_ group (values ranging between 15.33 ± 1.61 and 22.17 ± 2.16 mm), while the G.EE_35_ group had no inhibitory effect against this enterobacterium (Supplementary Table S1 and Figure 4). Considering the Zone Diameter Interpretive Standards for each antibiotic–microorganism [39] (Figure 4), *E. coli* was revealed to be also susceptible to the action of AMC and resistant to ERY.

Considering the inhibition diameters displayed by the G.EE_70_ group (Supplementary Table S1 and Figure 4), only the mG.EE_70_ extract displayed an inhibition zone whose diameter was similar to the value obtained for amoxicillin/clavulanic acid (ANOVA_1w_, F_(6,14)_ = 80.3, *p* < 0.001; Figure 4) and even exceeded the reference susceptibility value for this antibiotic (green line, Figure 4). Concluding, although not statistically significant, the mixture mG.EE_70_ was proved to be once again the most potent extract, this time against *E. coli.*

## 3. Discussion

The steady increase in antimicrobial resistance worldwide has become a significant public health concern recently recognized by the World Health Organization [3,41]. In response, there has been a growing effort in recent years to identify and validate new natural and multitargeting approaches as potential replacements for commercial antibiotics given their limited effectiveness against pathogenic microorganisms. Antimicrobial activity has been described for several samples of Portuguese propolis—from Gerês [23,25,28], Caramulo [42], Trás-os-Montes [43], Açores [44], and Bragança and Beja [45]. However, a direct comparison with commercial antibiotics was never reported. Therefore, in this study, we evaluated the antimicrobial potential of extracts of Gerês propolis collected over five consecutive years (G11–G15), as well as of the extract of the mixture (mG.EE), along with the efficacy of three commercial antibiotics—erythromycin, vancomycin, and amoxicillin/clavulanic acid.

The most frequent experimental approaches to estimate the antimicrobial effect of propolis are the disk diffusion and the agar dilution methodologies, either in solid or liquid media [18,46]. Each method can be particularly suitable in certain experimental conditions when chosen accordingly [47]. In this case, the agar dilution methodology was selected to test the extract samples prepared with ethanol 70%, more precisely G11.EE_70_–G15.EE_70_ and mG.EE_70_. These propolis extracts lacked antifungal capacity and did not affect MRSA and *E. coli* (Table 1). This aligns with previous studies, which reported no antifungal activity and a lower antibacterial potential against Gram-negative bacteria like *E. coli* [24,25]. Conversely, and as previously published [46,48], ethanol extracts from Gerês propolis are more effective against Gram-positive bacteria. Our results are also consistent with the literature for other extracts of Gerês propolis [22,25,28], although the extracts in the literature were prepared with ethanol 100% instead of ethanol 70%, as all the tested G.EE_70_ samples were effective against *B. subtilis* (MIC value = 50 μg/mL) and MSSA (MIC value = 200 μg/mL) (Table 1). Indeed, *B. subtilis* was the most susceptible strain. Beyond this antibacterial consistency with the antimicrobial spectra of other G.EE_100_ samples, no differences were observed within the antimicrobial activity of the different hydroalcoholic extracts. Therefore, we decided to conduct the disk diffusion assay, aiming to better characterize and distinguish the activity of each G.EE against the selected susceptibility strain panel.

The disk diffusion method was selected to screen a high number of samples, more precisely, 12 extracts (G11.EE_35_, G12.EE_35_, G13.EE_35_, G14.EE_35_, G15.EE_35_, G11.EE_70_, G12.EE_70_, G13.EE_70_, G14.EE_70_, G15.EE_70_, mG.EE_35_, and mG.EE_70_), and to quickly analyze their potential and effectiveness. Additionally, an antibiotic susceptibility test was conducted using antibiotic disks [49]. The results were presented as the diameter of the inhibition halos (Supplementary Table S1). Overall, the disk diffusion assay proved to be the most suitable approach for our study.

None of the Gerês propolis samples extracts (G.EE_70_ and G.EE_35_ groups) showed antifungal capacity against the tested yeasts: *C. albicans* and *S. cerevisiae*. Previous studies demonstrated that the same propolis sample from Gerês extracted with ethanol absolute (100%) had a small inhibition effect against yeasts (MIC = 1500 μg/mL) [28]. These differences can possibly be explained by the chosen solvent, which interferes with the chemical composition of the extracts [50,51]. Indeed, a higher percentage of ethanol culminates in different and/or more phenolic and flavonoid compounds, which are usually associated with an increased antifungal capacity [52,53].

Regarding the antibacterial potential, Gerês propolis extracts displayed a concentration-dependent antibacterial effect, with the most promising results observed in the G.EE_70_ group (Supplementary Table S1). The tested G.EEs exhibited evident activity against Gram-positive bacteria and a weaker effect towards Gram-negative species (Supplementary Table S1), namely, *E. coli* (MIC > 2000 μg/mL), corroborating previously published data [9,18,22,25,28,46]. *E. coli* strains can have different impacts on human health; some of them are harmless and beneficial to humans, while others can lead to a range of illnesses, spanning from mild and self-limiting gastroenteritis to more severe conditions like renal failure and septic shock [44]. Given the rising number of infections caused by this bacterium, there is an increasing need to explore novel approaches to combat this microbial threat. Our results against *E. coli* revealed that mG.EE_70_ (inhibition diameter of 22.17 ± 2.16 mm) presented a similar inhibitory effect to the amoxicillin/clavulanic acid (Figure 4; inhibition diameter of 22.67 ± 1.53 mm), the only commercial antibiotic that affected the Gram-negative bacterium and whose mode of action relies on inhibiting bacterial cell wall synthesis [54]. This finding suggests that effective action against *E. coli* is achievable with higher extract concentration/ethanol proportions. Additionally, our results revealed that mixing propolis samples can enhance the antimicrobial potential of the individual extracts, which might be attributed to a potential synergistic effect among compounds that are differently present in the individual extracts [55], although such synergy was not observed in the calculation of MIC values (Table 1). Recently, Peixoto et al. [27,28] highlighted how interesting the extracts of mixtures of propolis samples are since combining extracts of propolis from Gerês with different levels of bioactivities resulted in comparable and even higher bioactivities. Indeed, these natural mixtures could be a possible way of applying propolis in the future while overcoming the problems of standardization and scarcity. Overall, mixtures of propolis extracts and/or extracts of propolis blends from Gerês may be a promising alternative to antibiotics in combating Gram-negative bacteria, given the results against *E. coli*, and addressing the challenges posed by their distinct cell wall structure and antibiotic resistance.

Analyzing now the activity of G.EEs against the Gram-positive bacteria—*B. subtilis*, MSSA, and MRSA—it becomes evident that extracts prepared with ethanol 70% had comparable or even superior antimicrobial capacity when compared to certain antibiotics (Supplementary Table S1). More specifically, G.EE_70_ samples displayed greater activity against *B. subtilis* (values ranging between 25.25 ± 2.46 and 31.92 ± 4.48 mm; MIC = 50 μg/mL) and *S. aureus* (values ranging between 26.93 ± 2.20 and 29.17 ± 2.75 mm; MIC = 200 μg/mL) than vancomycin (inhibition diameter of 20.08 ± 0.12 and 16.50 ± 0.71 mm, respectively), an extensively utilized and long-standing antibiotic known for its higher potential for Gram-positive bacteria [56]. Vancomycin is a glycopeptide commercial antibiotic that exerts its bactericidal effect by blocking the polymerization of peptidoglycans in the bacterial cell wall [56]. The resistance mechanism of Gram-positive microorganisms against this antibiotic involves alterations in the bacterial cell wall structure through specific gene mutations or the acquisition of resistance genes from other bacteria [19,57]. In light of this, it is plausible to infer that, if propolis extracts exhibit increased antimicrobial effectiveness compared to vancomycin, the mechanism of action of propolis in this specific type of bacteria is a distinct (and more effective) pathway. This outcome emphasizes the potential of Gerês propolis extracts as a potent and more natural substitute for vancomycin in treating Gram-positive bacterial infections. Lastly, it is important to note that the antimicrobial effect of G.EEs against *S. aureus* (Table 1, Figure 2 and Supplementary Table S1), more specifically, MSSA (MIC = 200 μg/mL and inhibition diameter = 26.93–29.17 mm), supports the evidence that propolis from the north and center of Portugal is exceedingly efficient against this microorganism [45], which is currently one of the six leading mortality-causing pathogens [58], making this result extremely important for human health maintenance. Indeed, *S. aureus* is notorious for its capacity to become resistant to antibiotics, reinforcing the importance of the previous result [59,60].

MRSA is a strain of *S. aureus* resistant to methicillin and other β-lactam antibiotics commonly used for treating bacterial infections [61,62]. This resistance is due to the mecA gene, which encodes penicillin-binding protein 2a (PBP2a) within the staphylococcal cassette chromosome mec [63]. MRSA is a major multidrug-resistant pathogen, posing a significant health threat due to its strong resistance, ability to cause severe skin and hospital-acquired infections, and rapid spread in healthcare settings and communities [35,36,37]. Regarding our results, this microorganism was revealed to be resistant to all the tested commercial antibiotics (no growth inhibition zones) (Supplementary Table S1 and Figure 3). However, although MIC was not determined under the experimental conditions tested herein (MIC > 2000 μg/mL), the G.EE_70_ group exhibited a marked effect against MRSA (inhibition diameter = 17.25–21.33 mm) (Table 1; Supplementary Table S1; and Figure 3), suggesting that propolis from Gerês can be an important source of antimicrobial compounds or lead to the development of antimicrobial drugs against this bacterium. As the mode of action of each antibiotic is specific for a single cellular mechanism—erythromycin is an inhibitor of protein synthesis [64]; vancomycin is an inhibitor of bacterial cell wall synthesis [56]; and amoxicillin/clavulanic acid is an inhibitor of the bacterial wall combined with an inhibitor of *β*-lactamase [65]—the antibacterial effect of the G.EEs might be related to their chemical diversity, which confers to propolis the possibility to act in different cellular mechanisms. Indeed, several authors [25,55,66] suggested that bioactivities of propolis might be associated with synergistic effects between chemical compounds such as phenylpropanoids, p-coumaric acid, and diterpenic acid [55]. Regarding the G.EE_35_ group, all the tested G.EE_35_ samples did not exhibit an inhibitory effect against *S. aureus*—either MSSA or MRSA—demonstrating that the dilution of extracts and consequently the number of propolis compounds may limit the propolis activity against MRSA.

The chemical composition of propolis is highly dependent on the primary plant source, the season, and the bee species [9]. Consequently, its composition is known for being extremely diverse, complex, and variable [7,9,10]. Propolis is rich in phenolic compounds, particularly in flavonoids and aromatic acids, which are closely associated with its pharmacological properties, including antimicrobial capacity [67,68]. The total phenolic and flavonoid content reported for the six hydroalcoholic extracts (70% ethanol) analyzed in this study—G11.EE_70_, G12.EE_70_, G13.EE_70_, G14.EE_70_, G15.EE_70_, and mG.EE_70_—ranged from 71.00 ± 13.31 to 112.86 ± 19.24 mg GAE/g and from 40.73 ± 5.25 to 57.55 ± 6.69 mg QE/g, respectively [29]. Araújo et al. [29] further illustrated that diluting ethanol extracts from Gerês propolis significantly reduces their phenolic and flavonoid contents. The total phenolic and flavonoid content reported for G.EE_35_ samples and the mG.EE_35_ ranged between 67.61 ± 7.68 and 101.36 ± 10.81 mg GAE/g and between 30.49 ± 3.85 and 38.43 ± 5.72 mg QE/g, respectively [29]. This decrease in the TPC and TFC content affects extracts’ antimicrobial capacity (Figure 1 and Figure 2), underscoring the crucial role of these chemicals in propolis bioactivity. They also observed that these Gerês propolis blends exhibited TPC and TFC values surpassing the average values obtained from the individual hydroalcoholic extracts. Considering the known connection between these compounds and the pharmacological properties of propolis [67], it is reasonable to speculate that the heightened antimicrobial efficacy of G.EE_70_ samples (Supplementary Table S1) could be attributed to the greater abundance of flavonoids and phenolics in these hydroalcoholic extracts. Previous studies showed that the main phenolic compounds in Gerês propolis are apigenin, pinobanksin, chrysin, acacetin, galangin, kaempferide, kaempferol, caffeic acid, caffeic acid isoprenyl ester (CAIE), 3,4-dimethyl-caffeic acid (DMCA), p-coumaric acid, p-coumaric acid methyl ester, and ferulic acid [13,22]. Additionally, these studies demonstrated that these compounds are consistently present in propolis samples collected over consecutive years [13,22]. Therefore, considering this constancy of chemical profile observed in ethanol extracts of Gerês propolis [22], along with the relatively consistent antimicrobial spectra of the tested G.EEs, it is feasible to conclude that the G11.EEs–G15.EEs could have a phenolic composition similar to that already described for this type of propolis. In summary, our antimicrobial findings align with the documented chemical composition and known bioactive compounds of the tested hydroalcoholic extracts, emphasizing that higher TFC and TPC values lead to greater antimicrobial potential in this type of propolis.

Overall, and considering all outcomes, it is possible to conclude that propolis extracts are potent antimicrobial agents [69]. Moreover, these extracts represent a compelling and promising alternative, either as the natural product itself or as a source of compounds (lead) for commercial antibiotics, addressing the growing concern of antimicrobial resistance observed in recent years. Araújo et al. [29] also provided evidence of the in vivo and in vitro anti-inflammatory capacity of the same G.EEs used in this work. Thus, this combination of antimicrobial and anti-inflammatory potential underscores the biomedical value of G11–G15 propolis extracts—or the even greater potential of the extract from the mixture of the propolis samples G11, G12, G13, G14, and G15—and emphasizes the importance of further extensive research on Portuguese propolis from Gerês.

## 4. Materials and Methods

### 4.1. Propolis Samples

Propolis was harvested in an apiary located next to the Cávado River, between the settlements of Paradela and Sirvozelo, in Montalegre, Gerês, Portugal (41°45′41.62″ N; 7°58′03.34″ W). Raw propolis samples were collected over five years (2011–2015) and identified with the nomenclature criteria adopted in our research group as G11, G12, G13, G14, and G15—samples were named with the capital letter G (referring to their provenance: Gerês) followed by the last two digits of the harvesting year. Propolis samples (G11–G15) were kept at 4 °C for 5, 4, 3, 2, and 1 years, respectively.

### 4.2. Preparation of Propolis Hydroalcoholic Extracts

Hydroalcoholic extraction of raw propolis was performed according to the methods of previous studies [29]. Succinctly, 10 mL of ethanol 70% (*v*/*v*) (analytical ACS grade (Sigma-Aldrich, St. Louis, MO, USA)) was added individually to each propolis sample—G11, G12, G13, G14, and G15—and incubated under orbital agitation at 100 rpm and 25 °C, in the dark, for 24 h. Suspensions were then centrifuged for 5 min at 5000 rpm and 4 °C. The resultant supernatant was gathered and reserved, and the pellets were extracted again under the same conditions. After the second centrifugation, the resultant supernatant was combined with the previous one, generating stock solutions of 80 mg/mL of each G.EE_70_. Hydroalcoholic extracts with a final 40 mg/mL concentration were prepared by diluting G.EE_70_ samples in water (1:1), generating the G.EE_35_ samples. The percentage of solvent (ethanol 70% or 35%) was added to each code to help identify the ten G.EEs: G11.EE_70_ and G11.EE_35_; G12.EE_70_ and G12.EE_35_; G13.EE_70_ and G13.EE_35_; G14.EE_70_ and G14.EE_35_; G15.EE_70_ and G15.EE3_35_.

Hydroalcoholic extraction of the combination of all propolis samples from Gerês (mG) was carried out as stated by Araújo et al. [29]. In a nutshell, 125 mL of ethanol 70% (*v*/*v*) was incubated with G11, G12, G13, G14, and G15 (6.70 g, 6.88 g, 6.57 g, 6.57 g, and 6.67 g, respectively, taking into consideration the extraction yields previously obtained [29]). The mixture was swirled orbitally at 100 rpm for 24 h, in the dark and centrifuged, and the pellet was subsequently re-incubated with ethanol 70% under the conditions mentioned. The resultant supernatants were pooled, giving a stock solution of 80 mg/mL of mG.EE_70_. Additionally, mG.EE_35_ was prepared by diluting mG.EE_70_ in water (1:1).

### 4.3. Evaluation of Propolis Antimicrobial Potential

#### 4.3.1. Strains and Culture Conditions

Three Gram-positive bacteria—*Bacillus subtilis* 48886, methicillin-sensitive *Staphylococcus aureus* ATCC 6538 (MSSA), and methicillin-resistant *Staphylococcus aureus* M746665 (MRSA)—and one Gram-negative bacterium—*Escherichia coli* CECT 423—as well as the yeasts *Candida albicans* 53B and *Saccharomyces cerevisiae* BY4741, all from the microbial collection of the Department of Biology of the University of Minho, were selected as susceptibility indicator strains.

Bacteria were cultured in LB broth (Difco—composed of 0.5% (*w*/*v*) yeast extract, 1% (*w*/*v*) tryptone, and 2% (*w*/*v*) NaCl) or in solid LB medium (LBA—LB recipe with 2% (*w*/*v*) agar (Biolife, Milano, Italy)). Bacterial cultures were prepared using LB medium at a ratio of 2:5 (*w*/*v*), and incubation was performed at 37 °C and 200 rpm.

Yeasts were cultured in YPD medium (Difco^TM^ YPD Broth BD—composed of 1% (*w*/*v*) yeast extract, 2% (*w*/*v*) peptone, and 2% (*w*/*v*) dextrose/glucose) or in solid YPD medium (YPDA—YPD recipe with 2% (*w*/*v*) agar). Yeast cultures were prepared using YPD medium at a ratio of 2:5 (*w*/*v*), and incubation was conducted at 30 °C and 200 rpm. Microbial growth was monitored by determining the optical density at 600 nm (OD_600_) (Thermo Scientific Genesys 20 (Thermo Fischer Scientific, Waltham, MA, USA)).

#### 4.3.2. Antimicrobial Potential of Gerês Propolis: The Agar Dilution Assay

The antimicrobial capacity of propolis extracts from Gerês was evaluated by calculating the minimum inhibitory concentration (MIC) values of 6 hydroalcoholic extracts—G11.EE_70_, G12.EE_70_, G13.EE_70_, G14.EE_70_, G15.EE_70_, and mG.EE_70_—using an adaptation of the agar dilution method [66]. Briefly, yeast and bacterial strains were grown on YPD and LB media (see Section 4.3.1), respectively. Overnight cultures were diluted with fresh medium to an OD_600_ of 0.1 and incubated to the mid-exponential phase of growth (OD_600_ between 0.4–0.6), being 5 μL-drops of each culture inoculated on YPDA or LBA plates containing the propolis extracts at concentrations of 10, 50, 100, 200, 500, 750, 1000, 1500, and 2000 μg/mL. Plates with only LBA and YPDA, and with LBA and YPDA supplemented with ethanol 70% in the same volumes as the extracts, were used as controls. The control using LBA/YPDA alone was included to manage medium contaminations and confirm microbial growth, whereas the control with LBA/YPDA supplemented with ethanol was included to eliminate the influence of the extraction solvent on the observed results. Plates were incubated at 30 °C (Heraeus Incubator (Thermo Fischer Scientific, Waltham, MA, USA)) for 48 h for yeast, and at 37 °C (Incucell MMM MedCenter Incubator, Planegg, Germany) for 24 h in the case of bacteria, and then observed for the presence or absence of growth. MIC values were expressed as the lowest concentrations where no growth was verified.

#### 4.3.3. Antimicrobial Potential of Gerês Propolis: The Disk Diffusion Assay

The antimicrobial activity of propolis from Gerês was evaluated by measuring the diameter of the inhibition zones caused by G.EEs and mG.EEs against the panel of selected microbial indicator strains using an adaptation of the disk diffusion assay [39,70,71]. Three commercial antibiotics—amoxicillin/clavulanic acid (AMC), erythromycin (ERY), and vancomycin (VAN)—were also used for comparison purposes. Bacteria are considered susceptible to the tested antibiotics when the diameter of the inhibition zone is ≥23 mm in the case of ERY; ≥15 mm for VAN; and ≥27 mm (for *Staphylococcus* spp.) or ≥18 mm (for other bacteria) in the case of AMC [33].

Succinctly, mid-exponentially growing yeast and bacterial cultures were obtained as described in Section 4.3.1. A volume of 200 μL of each suspension was then added to 10 mL of 0.8% (*w*/*v*) agar (Biolife, Bothell, WA, USA) and poured on top of an LBA or YPDA plate, depending on the tested microorganism, forming an overlay. Blank paper disks (Thermo Scientific Oxoid^TM^, Hampshire, UK) were placed on top of these microbial layers, and 50 μL of each propolis sample—G.EE_70_ samples/mG.EE_70_ at 80 mg/mL and G.EE_35_ samples/mG.EE_35_ at 40 mg/mL—was added to an individual disk. Antibiotic-containing disks—AMC 30 μg, ERY 15 μg, and VAN 30 μg (BD BBL^TM^ Sensi-Disc^TM^, Porto, Portugal)—were also added on top of the formed microbial layer. Blank paper disks with 50 μL of each solvent (ethanol 70% and 35%) were used as controls. Plates were incubated at 30 °C for 48 h for yeasts, and at 37 °C for 24 h in the case of bacteria, followed by observation of the presence of inhibitions halos and measurement of the respective diameters (in mm) using a ruler.

### 4.4. Statistical Analysis

Unless otherwise stated experiments, were performed at least three times, each with three replicates per treatment. All data are presented as means ± SD (standard deviation). The statistical analysis was performed using GraphPad Prism 7 software (GraphPad Software, Inc, New York, NY, USA). Differences between results were evaluated using an ANOVA (one-way analysis of variance) followed by a t-test with Bonferroni correction for multiple comparisons. Only the analyses with a *p* < 0.05 were considered statistically significant (* 0.05 > *p* ≥ 0.01, ** 0.01 > *p* ≥ 0.001, *** *p* < 0.001).

## 5. Conclusions

Portuguese propolis from Gerês displays a wide spectrum of bioactivities, making it extremely appealing for several applications, namely, in the medicinal and health sector as an alternative or complement to non-efficient drugs. The G11–G15 hydroalcoholic propolis extracts and resultant mixtures (mG.EEs) (70% ethanol and 35% ethanol) demonstrated a potential antimicrobial capacity against the evaluated pathogens. Gerês propolis extracts prepared with ethanol 70% demonstrated higher antimicrobial potential (larger growth inhibition zones) compared to the tested antibiotics and are thus a promising option for treating infections, especially those caused by the methicillin-resistant *S. aureus* pathogen and Gram-negative bacteria, such as *E. coli*. On the other hand, the G.EE_35_ samples exhibited lower antibacterial activity compared to the G.EE_70_ samples and the tested antibiotics, leading us to deduce that the dilution process could be a constraining factor. This process may result in reduced phenolic and flavonoid contents, thereby potentially decreasing the antibacterial effectiveness of the G.EE_35_ extracts. Furthermore, we provided compelling evidence showcasing the extremely promising application of propolis mixtures as an alternative to individual extracts. Mixtures of Gerês propolis extracts resulted in a synergistic enhancement of antibacterial effectiveness, enabling us to take full advantage of the biomedical potential of Portuguese propolis from Gerês.

We provided further evidence of the potential application of Portuguese propolis as a natural alternative to commercial antibiotics in order to overcome the antimicrobial resistance challenge. We strongly advocate for further exploration of this natural product as a valuable biomedical agent or as a source of compounds with significant biomedical potential.

## Figures and Tables

**Table 1:** MIC values of Gerês propolis hydroalcoholic extracts against susceptibility indicator strains. Propolis extracts have ethanol 70% (*v*/*v*) (EE_70_). Results are expressed as the lowest concentration at which no growth occurred. Controls were performed using YPDA/LBA plates without G.EEs and YPDA/LBA plates with ethanol 70%. (G: Gerês; mG: a mixture of propolis samples from Gerês).

	*Bacillus subtilis*	Methicillin-Sensitive *Staphylococcus aureus* (MSSA)	Methicillin-Resistant *Staphylococcus aureus* (MRSA)	*Escherichia coli*
G11.EE_70_	50	200	>2000	>2000
G12.EE_70_	50	200	>2000	>2000
G13.EE_70_	50	200	>2000	>2000
G14.EE_70_	50	200	>2000	>2000
G15.EE_70_	50	200	>2000	>2000
mG.EE_70_	50	200	>2000	>2000

## Data Availability

Data are contained within the article and Supplementary Materials.

## Supplementary Materials

The following supporting information can be downloaded at: https://www.mdpi.com/article/10.3390/antibiotics13070655/s1, Table S1: Antimicrobial effect of propolis hydroalcoholic extracts from Gerês.

## References

[1] Acar J., Röstel B. (2001). Antimicrobial resistance: An overview. Rev. Sci. Tech..

[2] Hofer U. (2019). The cost of antimicrobial resistance. Nat. Rev. Microbiol..

[3] Salatino A. (2022). Perspectives for Uses of Propolis in Therapy against Infectious Diseases. Molecules.

[4] Davies J., Davies D. (2010). Origins and Evolution of Antibiotic Resistance. Microbiol. Mol. Biol. Rev..

[5] Murray C.J.L., Ikuta K.S., Sharara F., Swetschinski L., Robles Aguilar G., Gray A., Han C., Bisignano C., Rao P., Wool E. (2022). Global burden of bacterial antimicrobial resistance in 2019: A systematic analysis. Lancet.

[6] OECD (2018). Stemming the Superbug Tide: Just a Few Dollars More.

[7] Marcucci M.C. (1995). Propolis: Chemical composition, biological properties and therapeutic activity. Apidologie.

[8] Umthong S., Phuwapraisirisan P., Puthong S., Chanchao C. (2011). In vitro antiproliferative activity of partially purified Trigona laeviceps propolis from Thailand on human cancer cell lines. BMC Complement. Altern. Med..

[9] Silva-Carvalho R., Baltazar F., Almeida-Aguiar C. (2015). Propolis: A Complex Natural Product with a Plethora of Biological Activities That Can Be Explored for Drug Development. Evid.-Based Complement. Altern. Med..

[10] Kasote D., Bankova V., Viljoen A.M. (2022). Propolis: Chemical diversity and challenges in quality control. Phytochem. Rev..

[11] Moreira L., Dias L.G., Pereira J.A., Estevinho L. (2008). Antioxidant properties, total phenols and pollen analysis of propolis samples from Portugal. Food Chem. Toxicol..

[12] Šuran J., Cepanec I., Mašek T., Radić B., Radić S., Tlak Gajger I., Vlainić J. (2021). Propolis Extract and Its Bioactive Compounds—From Traditional to Modern Extraction Technologies. Molecules.

[13] Caetano A.R., Oliveira R.D., Celeiro S.P., Freitas A.S., Cardoso S.M., Gonçalves M.S.T., Baltazar F., Almeida-Aguiar C. (2023). Phenolic Compounds Contribution to Portuguese Propolis Anti-Melanoma Activity. Molecules.

[14] Widelski J., Okińczyc P., Suśniak K., Malm A., Paluch E., Sakipov A., Zhumashova G., Ibadullayeva G., Sakipova Z., Korona-Glowniak I. (2023). Phytochemical Profile and Antimicrobial Potential of Propolis Samples from Kazakhstan. Molecules.

[15] Popova M.P., Chinou I.B., Marekov I.N., Bankova V.S. (2009). Terpenes with antimicrobial activity from Cretan propolis. Phytochemistry.

[16] Krzyżek P., Paluch E., Gościniak G. (2020). Synergistic Therapies as a Promising Option for the Treatment of Antibiotic-Resistant Helicobacter pylori. Antibiotics.

[17] Akilandeswari K., Ruckmani K. (2016). Synergistic antibacterial effect of apigenin with β-lactam antibiotics and modulation of bacterial resistance by a possible membrane effect against methicillin resistant *Staphylococcus aureus*. Cell. Mol. Biol..

[18] Fokt H., Pereira A., Ferreira A., Cunha A., Almeida Aguiar C. (2010). How do bees prevent hive infections? The antimicrobial properties of propolis. Curr. Res. Technol. Educ. Top. Appl. Microbiol. Microb. Biotechnol..

[19] Reygaert W.C. (2018). An overview of the antimicrobial resistance mechanisms of bacteria. AIMS Microbiol..

[20] Baran A., Kwiatkowska A., Potocki L. (2023). Antibiotics and Bacterial Resistance—A Short Story of an Endless Arms Race. Int. J. Mol. Sci..

[21] Anjum S.I., Ullah A., Khan K.A., Attaullah M., Khan H., Ali H., Bashir M.A., Tahir M., Ansari M.J., Ghramh H.A. (2019). Composition and functional properties of propolis (bee glue): A review. Saudi J. Biol. Sci..

[22] Freitas A.S., Cunha A., Cardoso S.M., Oliveira R., Almeida-Aguiar C. (2019). Constancy of the bioactivities of propolis samples collected on the same apiary over four years. Food Res. Int..

[23] Freitas A.S., Cunha A., Parpot P., Cardoso S.M., Oliveira R., Almeida-Aguiar C. (2022). Propolis Efficacy: The Quest for Eco-Friendly Solvents. Molecules.

[24] Freitas A.S., Costa M., Pontes O., Seidel V., Proença F., Cardoso S.M., Oliveira R., Baltazar F., Almeida-Aguiar C. (2022). Selective Cytotoxicity of Portuguese Propolis Ethyl Acetate Fraction towards Renal Cancer Cells. Molecules.

[25] Freitas A.S., Cunha A., Oliveira R., Almeida-Aguiar C. (2022). Propolis antibacterial and antioxidant synergisms with gentamicin and honey. J. Appl. Microbiol..

[26] Oliveira R.D., Celeiro S.P., Barbosa-Matos C., Freitas A.S., Cardoso S.M., Viana-Pereira M., Almeida-Aguiar C., Baltazar F. (2022). Portuguese Propolis Antitumoral Activity in Melanoma Involves ROS Production and Induction of Apoptosis. Molecules.

[27] Peixoto M., Freitas A.S., Cunha A., Oliveira R., Almeida-Aguiar C. (2022). Mixing Propolis from Different Apiaries and Harvesting Years: Towards Propolis Standardization?. Antibiotics.

[28] Peixoto M., Freitas A.S., Cunha A., Oliveira R., Almeida-Aguiar C. (2021). Antioxidant and antimicrobial activity of blends of propolis samples collected in different years. LWT.

[29] Araújo C., Oliveira R.D., Pinto-Ribeiro F., Almeida-Aguiar C. (2022). An Insight on the Biomedical Potential of Portuguese Propolis from Gerês. Foods.

[30] Silva-Carvalho R., Miranda-Gonçalves V., Ferreira A.M., Cardoso S.M., Sobral A.J.F.N., Almeida-Aguiar C., Baltazar F. (2014). Antitumoural and antiangiogenic activity of Portuguese propolis in in vitro and in vivo models. J. Funct. Foods.

[31] Errington J., van der Aart L.T. (2020). Microbe Profile: Bacillus subtilis: Model organism for cellular development, and industrial workhorse. Microbiology.

[32] Piggot P.J. (2009). Bacillus subtilis. Encyclopedia of Microbiology.

[33] Becton, Dickinson and Company (2020). BBLTM Sensi-DiscTM Antimicrobial Susceptibility Test Discs. https://dmec.moh.gov.vn/documents/10182/26822970/upload_00027222_1645284052129.pdf?version=1.0&fileId=26844772.

[34] Taylor T.A., Unakal C.G. (2023). Staphylococcus aureus Infection. StatPearls.

[35] Lee A.S., De Lencastre H., Garau J., Kluytmans J., Malhotra-Kumar S., Peschel A., Harbarth S. (2018). Methicillin-resistant *Staphylococcus aureus*. Nat. Rev. Dis. Prim..

[36] Turner N.A., Sharma-Kuinkel B.K., Maskarinec S.A., Eichenberger E.M., Shah P.P., Carugati M., Holland T.L., Fowler V.G. (2019). Methicillin-resistant *Staphylococcus aureus*: An overview of basic and clinical research. Nat. Rev. Microbiol..

[37] Stapleton P.D., Taylor P.W. (2002). Methicillin Resistance in *Staphylococcus aureus*: Mechanisms and Modulation. Sci. Prog..

[38] Mueller M., Tainter C.R. (2023). Escherichia coli Infection. StatPearls.

[39] Zaidan M.R.S., Noor Rain A., Badrul A.R., Adlin A., Norazah A., Zakiah I. (2005). In vitro screening of five local medicinal plants for antibacterial activity using disc diffusion method. Trop. Biomed..

[40] Nikaido H. (1989). Outer membrane barrier as a mechanism of antimicrobial resistance. Antimicrob. Agents Chemother..

[41] Levy S.B. (2022). Factors impacting on the problem of antibiotic resistance. J. Antimicrob. Chemother..

[42] Pereira L., Cunha A., Almeida-Aguiar C. (2022). Portuguese propolis from Caramulo as a biocontrol agent of the apple blue mold. Food Control.

[43] Dias L.G., Pereira A.P., Estevinho L.M. (2012). Comparative study of different Portuguese samples of propolis: Pollinic, sensorial, physicochemical, microbiological characterization and antibacterial activity. Food Chem. Toxicol..

[44] Lourenço T., Oliveira T., Ferreira A.M., Oliveira R., Bento F., Geraldo D., Almeida-Aguiar C., Cunha A. (2014). Antimicrobial and antioxidant properties of propolis ethanol extracts from Terceira Island (Azores, Portugal). Planta Med..

[45] Silva J.C., Rodrigues S., Feás X., Estevinho L.M. (2012). Antimicrobial activity, phenolic profile and role in the inflammation of propolis. Food Chem. Toxicol..

[46] Kujumgiev A., Tsvetkova I., Serkedjieva Y., Bankova V., Christov R., Popov S. (1999). Antibacterial, antifungal and antiviral activity of propolis of different geographic origin. J. Ethnopharmacol..

[47] Jorgensen J.H., Ferraro M.J. (2009). Antimicrobial Susceptibility Testing: A Review of General Principles and Contemporary Practices. Clin. Infect. Dis..

[48] Przybyłek I., Karpiński T.M. (2019). Antibacterial Properties of Propolis. Molecules.

[49] Truong W.R., Hidayat L., Bolaris M.A., Nguyen L., Yamaki J. (2021). The antibiogram: Key considerations for its development and utilization. JAC-Antimicrob. Resist..

[50] Sforcin J.M., Bankova V. (2011). Propolis: Is there a potential for the development of new drugs?. J. Ethnopharmacol..

[51] Cunha I.B.S., Sawaya A.C.H.F., Caetano F.M., Shimizu M.T., Marcucci M.C., Drezza F.T., Povia G.S., Carvalho P.O. (2004). Factors that influence the yield and composition of Brazilian propolis extracts. J. Braz. Chem. Soc..

[52] Al Aboody M.S., Mickymaray S. (2020). Anti-Fungal Efficacy and Mechanisms of Flavonoids. Antibiotics.

[53] Teodoro G.R., Ellepola K., Seneviratne C.J., Koga-Ito C.Y. (2015). Potential Use of Phenolic Acids as Anti-Candida Agents: A Review. Front. Microbiol..

[54] Singh N.S., Singhal N., Kumar M., Virdi J.S. (2021). Exploring the genetic mechanisms underlying amoxicillin-clavulanate resistance in waterborne *Escherichia coli*. Infect. Genet. Evol..

[55] Abdullah N.A., Ja’afar F., Yasin H.M., Taha H., Petalcorin M.I.R., Mamit M.H., Kusrini E., Usman A. (2019). Physicochemical analyses, antioxidant, antibacterial, and toxicity of propolis particles produced by stingless bee Heterotrigona itama found in Brunei Darussalam. Heliyon.

[56] Patel S., Preuss C.V., Bernice F. (2023). Vancomycin. StatPearls.

[57] Selim S. (2021). Mechanisms of gram-positive vancomycin resistance (Review). Biomed. Rep..

[58] Gajic I., Kabic J., Kekic D., Jovicevic M., Milenkovic M., Mitic Culafic D., Trudic A., Ranin L., Opavski N. (2022). Antimicrobial Susceptibility Testing: A Comprehensive Review of Currently Used Methods. Antibiotics.

[59] Grundmann H., Aires-de-Sousa M., Boyce J., Tiemersma E. (2006). Emergence and resurgence of meticillin-resistant *Staphylococcus aureus* as a public-health threat. Lancet.

[60] Chambers H.F., DeLeo F.R. (2009). Waves of resistance: *Staphylococcus aureus* in the antibiotic era. Nat. Rev. Microbiol..

[61] Bæk K.T., Gründling A., Mogensen R.G., Thøgersen L., Petersen A., Paulander W., Frees D. (2014). β-Lactam resistance in methicillin-resistant *Staphylococcus aureus* USA300 is increased by inactivation of the ClpXP protease. Antimicrob. Agents Chemother..

[62] Ali Alghamdi B., Al-Johani I., Al-Shamrani J.M., Musamed Alshamrani H., Al-Otaibi B.G., Almazmomi K., Yusnoraini Yusof N. (2023). Antimicrobial resistance in methicillin-resistant *Staphylococcus aureus*. Saudi J. Biol. Sci..

[63] Martínez-Meléndez A., Morfín-Otero R., Villarreal-Treviño L., González-González G., Llaca-Díaz J., Rodríguez-Noriega E., Camacho-Ortíz A., Garza-González E. (2015). Staphylococcal Cassette Chromosome mec (SCCmec) in coagulase negative staphylococci. Med. Univ..

[64] Weisblum B. (1995). Insights into erythromycin action from studies of its activity as inducer of resistance. Antimicrob. Agents Chemother..

[65] Todd P.A., Benfield P. (1990). Amoxicillin/Clavulanic Acid. Drugs.

[66] Stepanović S., Antić N., Dakić I., Švabić-Vlahović M. (2003). In vitro antimicrobial activity of propolis and synergism between propolis and antimicrobial drugs. Microbiol. Res..

[67] Wieczorek P.P., Hudz N., Yezerska O., Horčinová-Sedláčková V., Shanaida M., Korytniuk O., Jasicka-Misiak I. (2022). Chemical Variability and Pharmacological Potential of Propolis as a Source for the Development of New Pharmaceutical Products. Molecules.

[68] Miguel M.G., Nunes S., Dandlen S.A., Cavaco A.M., Antunes M.D. (2010). Phenols and antioxidant activity of hydro-alcoholic extracts of propolis from Algarve, South of Portugal. Food Chem. Toxicol..

[69] Vică M.L., Glevitzky M., Heghedus-Mîndru R.C., Glevitzky I., Matei H.V., Bâlici Ș., Popa M., Teodoru C.A. (2022). Potential Effects of Romanian Propolis Extracts against Pathogen Strains. Int. J. Environ. Res. Public Health.

[70] Bauer A.W., Kirby W.M.M., Sherris J.C., Turck M. (1966). Antibiotic Susceptibility Testing by a Standardized Single Disk Method. Am. J. Clin. Pathol..

[71] Wilkins T.D., Holdeman L.V., Abramson I.J., Moore W.E.C. (1972). Standardized Single-Disc Method for Antibiotic Susceptibility Testing of Anaerobic Bacteria. Antimicrob. Agents Chemother..

