# Supplementary material for: In Vitro Antimicrobial Potential of Portuguese Propolis Extracts from Gerês against Pathogenic Microorganisms
Source: Antibiotics (Basel). 2024 Jul 16;13(7):655. doi: 10.3390/antibiotics13070655 (PMC11273468; doi:10.3390/antibiotics13070655)
Supplement: Supplementary file 1 [file antibiotics-13-00655-s001.zip › antibiotics-2986556-supplementary.pdf]

antibiotics

Supplementary Material

Supplementary Table S1. Antimicrobial effect of propolis hydroalcoholic extracts from Gerês. Propolis extracts have ethanol 70% (v/v) (EE70) or 35% (EE35). Results are expressed as the mean value ± standard deviation (SD) of the diameter of the inhibition zones (mm) obtained for each propolis extract and commercial antibiotics erythromycin (ERY), vancomycin (VAN), and amoxicillin/clavulanic acid (AMC) against each tested strain. Negative controls were performed using blank paper disks with ethanol 70% and ethanol 35%. (G: Gerês; mG: a mixture of propolis samples from Gerês; mm: millimeter.)

	Bacillus subtilis		Methicillin-sensitive *Staphylococcus aureus* (MSSA)		Methicillin-resistant *Staphylococcus aureus* (MRSA)		*Escherichia coli*	
	**EE_{70}**	**EE_{35}**	**EE_{70}**	**EE_{35}**	**EE_{70}**	**EE_{35}**	**EE_{70}**	**EE_{35}**
**G11.EE**	30.71 ± 0.31	17.08 ± 2.31	27.58 ± 0.63	15.96 ± 0.75	18.75 ± 2.05	-	15.33 ± 1.61	-
**G12.EE**	31.92 ± 4.48	17.92 ± 2.01	29.17 ± 2.75	16.71 ± 1.48	17.25 ± 1.15	-	17.92 ± 0.72	-
**G13.EE**	25.25 ± 2.46	16.67 ± 3.62	28.47 ± 1.77	15.42 ± 1.45	18.92 ± 2.13	-	18.92 ± 1.61	-
**G14.EE**	28.21 ± 2.67	16.88 ± 2.07	27.03 ± 1.40	14.42 ± 0.14	19.75 ± 2.41	-	17.04 ± 1.63	-
**G15.EE**	30.32 ± 1.27	19.92 ± 2.72	26.93 ± 2.20	16.08 ± 2.28	17.50 ± 1.09	-	-	-
**mG.EE**	26.75 ± 2.71	17.25 ± 0.66	29.08 ± 1.63	18.08 ± 0.62	21.33 ± 3.17	-	22.17 ± 2.16	-
**ERY**	28.75 ± 1.77		22.50 ± 0.66		-		10.00 ± 0.00	
**VAN**	20.08 ± 0.12		16.50 ± 0.71		-		-	
**AMC**	13.63 ± 2.65		43.75 ± 0.66		-		22.67 ± 1.53	

"-" means no effect/absence of growth inhibition zone.
